# Residue Analysis and Dietary Risk Assessment of 10 Neonicotinoid Insecticides in *Oenanthe javanica* from Hainan Province of China

**DOI:** 10.3390/foods14010078

**Published:** 2024-12-31

**Authors:** Shusheng Tang, Zikang Zeng, Rui Kang, Ying Wei, Gaohao Tan, Minni Chen, Bin Wang, Zhuhua Tang

**Affiliations:** 1 National Key Laboratory of Veterinary Public Health and Safety, College of Veterinary Medicine, China Agricultural University, Beijing 100193, China; tssfj@cau.edu.cn (S.T.); zikangzeng@cau.edu.cn (Z.Z.); 2 Technology Innovation Center for Food Safety Surveillance and Detection (Hainan), Sanya Institute of China Agricultural University, Sanya 572025, China; 3 Key Laboratory of Tropical Fruits and Vegetables Quality and Safety, State Administration for Market Regulation, Hainan Academy of Inspection and Testing, Haikou 571199, China; 18789264887@163.com (R.K.); wy165084600@163.com (Y.W.); tangaohao2011@139.com (G.T.); 19907507977@163.com (M.C.)

**Keywords:** pesticide residues, *Oenanthe javanica*, neonicotinoid insecticides, risk assessment

## Abstract

In this study, residues of 10 neonicotinoid insecticides were tested with 143 fresh samples of *Oenanthe javanica* using the QuEChERS method combined with UPLC-MS/MS. Based on the residue results, the point estimation method was used to assess dietary risks for adults and children, and the cumulative risk was assessed according to the hazard index (*HI*) and relative potency factor (*RPF*) methods. The results showed that 71 out of 143 samples of fresh *Oenanthe javanica* sold in Hainan tested positive for neonicotinoid insecticides, with a detection rate of 49.65%. Six neonicotinoid insecticides were detected and their detection frequencies are as follows: imidacloprid (44.76%), acetamiprid (16.08%), clothianidin (13.29%), dinotefuran (4.90%), thiamethoxam (3.50%) and flonicamid (3.50%). The most frequently co-detected combination was imidacloprid and acetamiprid, with a detection rate of 29.58%. The results of acute and chronic dietary risks showed that all the obtained values of %*ADI* (acceptable daily intake) and %*ARfD* (acute reference dose) are far below 100%, so the risks of the six detected neonicotinoid insecticides are acceptable for various population groups. Meanwhile, the results of the cumulative risk showed that all the obtained values of the hazard index and the corrected total exposure concentration are far lower than the health guidelines, indicating that the residue levels of neonicotinoid insecticides in fresh *Oenanthe javanica* are acceptable. However, it is notable that its acute risk is higher than its chronic risk, and the dietary risk for children is higher than adults. These findings will provide a theoretical basis and objective data for supporting the revision of MRLs for *Oenanthe javanica*, as well as valuable guidance for the production, consumption, regulation and standardization of *Oenanthe javanica* sold in Hainan.

## 1. Introduction

*Oenanthe javanica*, commonly known as water celery, is a perennial aquatic herb in the Apiaceae family, widely distributed and cultivated across Asia and Oceania. This species is well suited to low-lying topography, including ponds and ditches from 600 to 4000 m. Research indicates that *Oenanthe javanica* is rich in a variety of bioactive compounds, including vitamins, phenolics, polysaccharides and alkaloids, which exhibit liver-protective, anti-inflammatory, antioxidant, antiviral and immune-enhancing effects [1,2].

However, during its growth, *Oenanthe javanica* is susceptible to infection by aphids and other pests, leading to an increasing reliance on pesticides to prevent pest damage and fungal infections [3]. Neonicotinoid insecticides (NEOs) are a class of insecticides that act on nicotinic acetylcholine receptors, thereby exerting unique neurotoxic effects. Due to their relatively low toxicity to mammals, they have become one of the most widely used types of insecticides. Nevertheless, research findings indicate that NEOs may lead to a reduction in wild bee populations [4]. The excessive use and long-term accumulation of these pesticides may result in chronic and cumulative toxicity in mammals, which can lead to a range of adverse effects, including decreased fertility, developmental delays, reduced mobility, deformities and an increased risk of diseases such as breast cancer [1,2]. The inappropriate use of pesticides can cause significant risks to both the ecological environment and consumer health, seriously threatening human well-being [5].

In 2014, the usage of NEOs accounted for over 25% of global insecticide consumption. These insecticides are registered in 120 countries and used on over 140 crops, particularly in Asia, America and Europe [6,7,8]. The registered NEOs in China include imidacloprid, thiamethoxam, acetamiprid, clothianidin, dinotefuran and chlorantraniliprole. The first five are commonly used on crops such as cowpea, cucumber, cabbage, peach, rice and cotton. Additional regulatory measures have been implemented intending to protect consumers and ecosystems. The Chinese government launched an annual program of quality and safety inspections of agricultural products in 2011 to ensure pesticide residues remain within safe limits.

Previous research in our laboratory has indicated that pesticide residues in single batches of *Oenanthe javanica* samples can range from one to twelve different pesticide types, where the amount of NEOs (imidacloprid, thiamethoxam and acetamiprid) is over 25% [9]. The current national standard, entitled the “National Food Safety Standard for Maximum Residue Limits of Pesticides in Food” (GB 2763-2021) [10], outlines the maximum residue limits (MRLs) for NEOs in vegetables, with a range of 0.01 to 20.00 mg/kg. However, no specific MRLs have been established for *Oenanthe javanica* or aquatic vegetables, and there is a notable lack of monitoring reports regarding pesticide residues in *Oenanthe javanica*. Therefore, it is important to carry out residue determination and risk assessment of NEOs in *Oenanthe javanica*. In this study, the QuEChERS method was used to extract and purify NEOs residues, then ultra-performance liquid chromatography–tandem mass spectrometry (UPLC-MS/MS) was used to analyze 143 samples of *Oenanthe javanica* sold in Hainan Province for 10 NEOs. Ultimately, the point estimation method was used to assess dietary risks for adults and children, and the cumulative risk was assessed according to the hazard index and relative potency factor methods. The findings of this study will provide a theoretical basis and objective data for supporting the revision of MRLs for *Oenanthe javanica*, as well as valuable guidance for the production and regulatory oversight of NEOs in *Oenanthe javanica*.

## 2. Materials and Methods

### 2.1. Chemical and Reagents

Imidacloprid, thiamethoxam, acetamiprid, clothianidin, thiacloprid, dinotefuran, nitenpyram, imidacloprid, flonicamid and sulfoxaflor were supplied by the Agro-Environmental Protection Institute of Ministry of Agriculture and Rural Affairs of China. Acetonitrile and ammonium formate were purchased from Merck KGaA., Darmstadt, Germany. Formic acid and methanol were purchased from Sigma-Aldrich Corp., St. Louis, MO, USA. Cleanert MAS-QuEChERS extraction kits was obtained from Agela Technologies., Tianjin, China, and the QuEChERS purification tube was obtained from KNORTH., Beijing, China.

### 2.2. Sample Collection and Preparation

A total of 143 samples (>2 kg/sample) of *Oenanthe javanica* were collected during the harvest season (November 2022–April 2023) in Hainan. After removing the roots, the whole samples were collected, then the samples were chopped and thoroughly homogenized. Finally, 100 g homogenates were sampled with the citrate-buffered CEN standard method and stored at −20 °C for further analysis.

After weighing 10 g of blank *Oenanthe javanica* samples, 50 µL and 100 µL of 10 mixed control intermediate solutions at a concentration of 2.0 µg/mL were added and a blank test was also conducted. A ceramic homogenizer was employed, and the mixture was shaken for 5 min to ensure thorough extraction. Then, the Cleanert MAS-QuEChERS was added into the mixture and vortexed for 30 s, followed by an additional vortexing (5 min). The sample was then centrifuged at 5000 rpm for 5 min. For purification, the supernatant was carefully transferred to the QuEChERS purification tube. The mixture was vortexed for 1 min and then centrifuged at 5000 rpm for 5 min. The supernatant was filtered through a 0.22 μm organic microporous membrane for UPLC-MS/MS analysis.

### 2.3. UPLC-MS/MS Analysis

#### 2.3.1. Liquid Chromatographic Conditions

Chromatographic column: ACQUITY UPLC^®^ HSS T3 (1.8 µm, 2.1 × 100 mm, Waters Corporation, Milford, CT, USA); mobile phase: A: 2 mmol/L ammonium formate—0.01% formic acid aqueous solution, B: 2 mmol/L ammonium formate—0.01% formic acid methanol solution; gradient elution procedure: 0–0.5 min 97% A (3% B), 0.5–7.0 min 97%–2% A (3%–98% B), 7.0–8.8 min 2% A (98% B), 8.8–9.0 min 2%–97% A (98%–3% B), 9.0–10.0 min 97% A (3% B); flow rate: 0.3 mL/min; column temperature: 40 °C; injecting volume: 1 µL; running time: 10 min.

#### 2.3.2. Mass Spectrometry Conditions

Ion source: electrospray ion source, simultaneous scanning in positive ion (ESI+) mode, detection mode: dynamic multiple reaction monitoring (DMRM); capillary voltage: 2.0 KV; ion source temperature: 150 °C; desolventisation temperature: 350 °C; desolventisation gas flow rate: 1000 L/h; conical pore gas flow rate: 150 L/h; collision gas flow rate: 0.11 mL/min; collision gas: argon. Other parameters are shown in Table 1.

#### 2.3.3. Instrumental Analysis

The determination of residues of 10 NEOs was conducted with UPLC-MS/MS (Xevo TQ-S, Waters, USA) according to the “National Food Safety Standard: Determination of 331 Pesticides and Metabolites Residues in Plant-Based Foods by Liquid Chromatography Mass Spectrometry” (GB 23200.121-2021) [11].

After adding 0.2 mL NEOs standard solution (100 µg/mL) into a 10 mL volumetric flask, a mixed standard stock solution with a concentration of 2 µg/mL for each pesticide was obtained through dilution with acetonitrile. The stock solution was stored in a dark place at −18 °C. With the stock solution and a blank matrix solution, the matrix-matched standard working solutions at concentrations of 0.002 mg/L, 0.005 mg/L, 0.01 mg/L, 0.02 mg/L, 0.05 mg/L, 0.1 mg/L and 0.2 mg/L were prepared to obtain the matrix-matched standard calibration curve. A matrix-matched standard curve was constructed using the chromatographic peak area of the quantifier ion as the y-axis and the concentration of the matrix-matched standard working solution as the x-axis. Relevant parameters of instrumental conditions are the same as our previous study [9].

### 2.4. Risk Assessment Methods

#### 2.4.1. Calculation of Acute and Chronic Dietary Risks

The point assessment method of the acceptable daily intake (*ADI*) and acute reference dose (*ARfD*) was used to assess the dietary risks of pesticides to specific populations. The specific population refers to adults and children 1 to 6 years of age and the average weights of Chinese adults and children aged 1 to 6 years were 53.23 kg and 16.14 kg [12], respectively. The chronic dietary risk was calculated according to Equations (1) and (2), where the *ADI* is divided by the estimated intake of *Oenanthe javanica* from daily food sources. A value of %*ADI* exceeding 100% indicates an unacceptable long-term exposure risk, whereas a value of 100% or below indicates an acceptable long-term exposure risk. The %*ARfD* represents the acute dietary risk, and it is calculated by Equation (3). When the *ARfD* value of the pesticides has been established, the short-term dietary risk can be calculated. A value of %*ARfD* exceeding 100% indicates an unacceptable level of risk. The lower value represents a lower level of risk.

(1) $$EDI={\;A}_{C}\;\times \;R_{C}\;\times \;\frac{Pi}{bw}$$

(2) $$\%ADI=\frac{EDI}{ADI}\times 100\%$$

(3) $$\%ARfD=\frac{LP\times HR}{bw\times ARfD}\times 100\%$$

In the above equations, *EDI* represents the estimated daily intake of a specific compound from food sources, expressed in mg/kg bw. *A_C_* represents the mean pesticide residue level detected in the tested samples of *Oenanthe javanica*, expressed in mg/kg. *R_C_* represents the estimated average daily intake of vegetables and edible fungi, based on the per-person consumption in Hainan Province, which is set at 0.287 kg/day according to the 2023 edition of the China Statistical Yearbook. *Pi* is the processing factor for *Oenanthe javanica* samples, set at 1. *bw* represents body weight, weighing 53.23 kg for adults and 16.14 kg for children [12]. *ADI* represents acceptable daily intake, expressed in mg/kg bw. *LP* represents large portion size, expressed in kg/d, representing the maximum daily consumption of a particular food item. For vegetables and edible fungi, the portion size is set at 0.42 kg, according to the China Statistical Yearbook (2023). *HR* represents the 99.9th percentile of pesticide residue values, whereas *ARfD* denotes the acute reference dose [13,14].

#### 2.4.2. Methods of Cumulative Exposure Assessment

The cumulative dietary risk for pesticides of the same category was calculated with the hazard index (*HI*) method and the relative potency factor (*RPF*) method [15]. The *HI* method was developed by the European Food Safety Authority to assess the cumulative risk of pesticides that produce the same toxic effect in organisms. The method can be used to calculate cumulative exposure risk for multiple pesticide residues, as shown in Equation (4).

(4) $$HI=\sum_{n=1}^{i}\%ADIn$$

In the *RPF* method, the corrected total exposure concentration (*TB_C_*, mg/kg) was calculated according to Equations (5)–(7) by converting the residue exposure concentrations of various NEOs found in vegetables into equivalent amounts of a reference chemical. Given the toxicity levels of different NEOs, the *TB_C_* of NEOs in *Oenanthe javanica* is assessed based on the *RPF* values recommended by the U.S. Environmental Protection Agency. Imidacloprid was chosen as the reference chemical for the *TB_C_* calculation because it is the most commonly used and it has the highest detection rate in this investigation. Meanwhile, imidacloprid exhibited the strongest toxicological evidence [13,14]. The chronic reference dose (*cRfD*) of imidacloprid, 0.057 mg/kg/d, is used as the reference value. If *TB_C_* < *cRfD* of imidacloprid, the dietary risk from NEOs residues is acceptable [13,16].

(5) $$E_{Ci}=B_{Ci}\times \frac{Rc}{bw}$$

(6) $${RPF}_{i}=\frac{{cRfD}_{imidacloprid}}{{cRfD}_{i}}$$

(7) $${TB}_{C}=\sum_{i=1}^{n}E_{Ci}\times {RPF}_{i}$$

In the above equations, *E_Ci_* represents the exposure concentration of the *i*-th pesticide residue in *Oenanthe javanica* (mg/kg/d). *B_Ci_* represents the average residue level (mg/kg). *R_C_* represents the estimated daily consumption of vegetables and edible fungi set at 0.287 kg/d. *bw* represents body weight, 53.23 kg for adults, 16.14 kg for children. *_C_RfD_i_* represents the chronic reference dose for the *i*-th NEOs. *TB_C_* represents the total combined exposure concentration of NEOs in vegetables (mg/kg/d), and *RPF_i_* represents the relative potency factor calculated based on the *cRfD* of imidacloprid.

### 2.5. Statistics

The data were presented as an average or standard error of the mean (SEM) and analyzed using GraphPadPrism 9.0 (GraphPad Software, La Jolla, CA, USA).

## 3. Results

### 3.1. Quality Control of the Experimental Process

The experimental results showed that the quality control for detecting 10 NEOs meets the requirements for pesticide residue analysis, as shown in Table 1. A good linear relationship was shown within the concentration range of 2 to 200 mg/L (*R*^2^ ≥ 0.99). The limit of detection (LOD) and limit of quantification (LOQ) were determined to be 3 µg/kg and 10 µg/kg, respectively, calculated based on the estimation method referenced from the “National Standard for Food Safety General Principles for the Validation of Chemical Analytical Methods” (GB 5009.295-2023) [17]. In this study, when the detected value was below LOD, the value of 1/2 LOD was defined. The range of recovery rates of spiked samples were 97.4% to 119.0% (10 μg/kg) and 94.6% to 120.0% (20 μg/kg), meeting the requirements of the “Code of Practice for Quality Control in the Laboratory Physical and Chemical Testing of Food” (GB 27404-2014) [18].

### 3.2. Residue Analysis of NEOs in Oenanthe javanica

**Figure 1:**
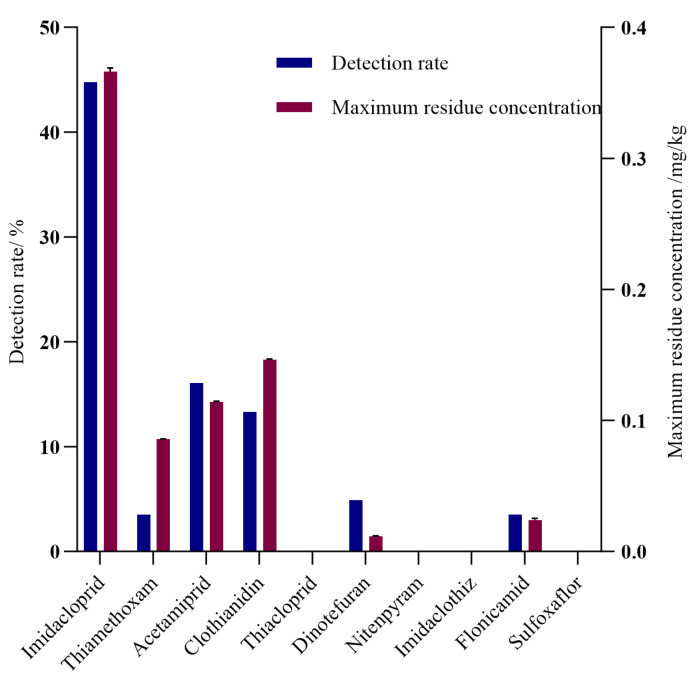
Levels and detection rates of pesticide residues in *Oenanthe javanica* sold in Hainan Province.

In this study, six NEOs were found in 71 samples out of 143 samples, resulting in a detection rate of 49.65%. The detected NEOs were imidacloprid, thiamethoxam, acetamiprid, dinotefuran, clothianidin and flonicamid. The detection rates of the pesticides, ranked from highest to lowest, were imidacloprid (44.76%) > acetamiprid (16.08%) > clothianidin (13.29%) > dinotefuran (4.90%) > thiamethoxam = flonicamid (3.50%), as shown in Figure 1. The highest detection value of imidacloprid was 0.37 mg/kg, with an average concentration of 0.028 mg/kg. Additionally, 11.89% of samples contained residues of three or more pesticides, 10.49% contained two pesticides and 29.37% contained one pesticide, with a maximum of six pesticides detected in a single sample. Imidacloprid and acetamiprid were the most frequent pesticide combinations, followed by imidacloprid and clothianidin. Specifically, 91.30% of the samples with acetamiprid also contained imidacloprid and 89.47% of the samples with clothianidin also contained imidacloprid. The most frequent combination of three pesticides was imidacloprid, acetamiprid and clothianidin, with 60.87% of samples containing acetamiprid also testing positive for imidacloprid and clothianidin, as shown in Table 2. Since no maximum residue limits (*MRLs*) for NEOs in *Oenanthe javanica* have been established, an exceedance rate could not be calculated.

### 3.3. Dietary Risk Assessment

**Figure 2:**
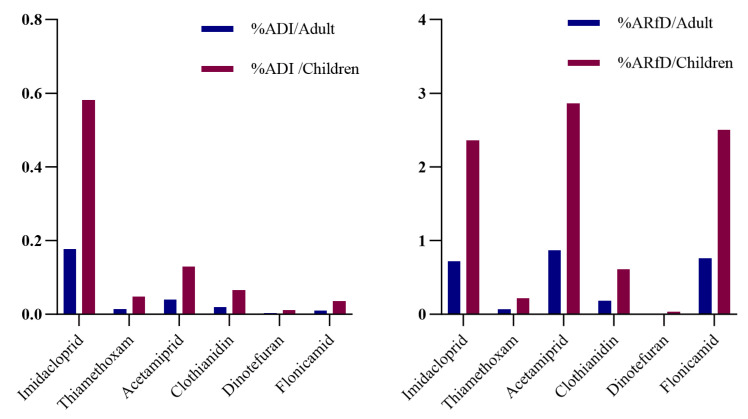
Risk assessment of acute and chronic dietary intake of pesticides on *Oenanthe javanica* sold in Hainan Province in different populations.

The acute and chronic dietary risk assessment results for the six detected NEOs in *Oenanthe javanica* are shown in Figure 2. For chronic dietary risk, the %*ADI* ranged from 0.0034% to 0.18% for adults and from 0.011% to 0.58% for children, where the highest value (0.58%) was significantly below 100%, indicating that the chronic dietary risk for the six pesticides was acceptable. For acute dietary risk assessment, the %*ARfD* ranges from 0.01% to 0.87% for adults and from 0.02% to 2.86% for children. The order from highest to lowest %*ARfD* was acetamiprid > flonicamid > imidacloprid > clothianidin > thiamethoxam > dinotefuran. Although acetamiprid had lower maximum residue levels than imidacloprid, its *ARfD* value was also lower, resulting in the highest %*ARfD* (2.86%) among the six pesticides. All the above values are far below 100%, suggesting that the acute dietary risk for the detected pesticides is acceptable, as shown in Table 3.

### 3.4. Cumulative Exposure Assessment

#### 3.4.1. HI Assessment

The *HI* values, the sum of the %*ADI* values for the six detected NEOs, were calculated to assess the cumulative risk according to Equation (4). The *HI* values for adults and children were 0.00264 and 0.00872, both of which are far less than 1. This indicates that the cumulative exposure risk of the six NEOs detected in the fresh *Oenanthe javanica* is acceptable.

#### 3.4.2. EPF Assessment

*TB_C_* was calculated according to Equations (5)–(7) as follows:

(8) $$\begin{array}{c} {TB}_{C}={EC}_{imidacloprid}\times 1.000+{EC}_{thiamethoxam}\times 9.500\\ +{EC}_{acetamiprid}\times 0.803+{EC}_{clothianidin}\times 5.816\\ +{EC}_{dinotefuran}\times 2.850+{EC}_{flonicamid}\times 1.425 \end{array}$$

The cumulative exposure concentrations for the six detected pesticides were 0.00054 mg/kg/d for adults and 0.00178 mg/kg/d for children, both of which are far less than the health guidance values. Among the pesticides, clothianidin had the highest exposure concentration of 0.00053 mg/kg/d for children, which is 0.93% of the health guidance value. This indicates that the dietary risk from NEOs in fresh *Oenanthe javanica* is low, and the safety risk is acceptable.

## 4. Discussion

### 4.1. Registration and Residue Status of 10 NEOs Insecticides in Oenanthe javanica

As of June 2024, *Oenanthe javanica* is classified as a stem-leaf vegetable within the aquatic vegetable category, according to GB 2763-2021. A review of the “China Pesticide Information Network” (www.chinapesticide.org.cn) shows that numerous pesticide products are registered for controlling aphids and rice planthoppers in crops such as rice and celery. However, there is currently no registration of NEOs used in *Oenanthe javanica*.

**Figure 3:**
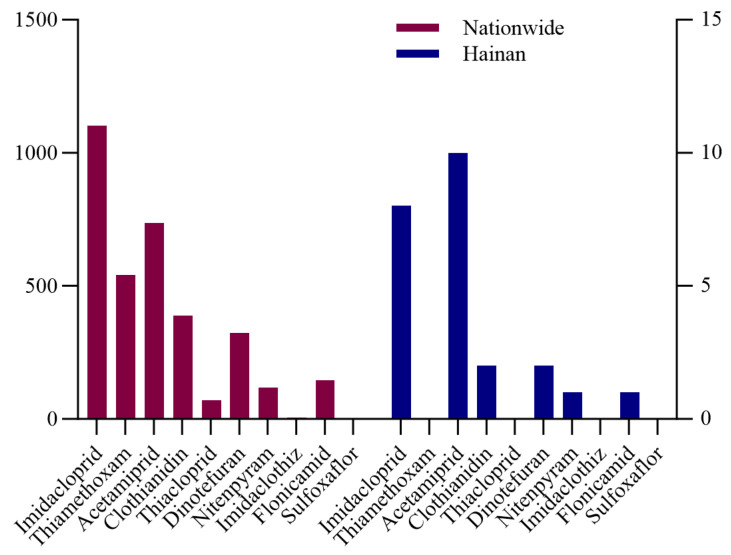
Registration of different NEOs.

In this study, 10 NEOs were detected, among which the most frequently detected one was imidacloprid, with a detection rate of 44.76% and the highest concentration of 0.37 mg/kg. This result is likely attributable to the frequency and volume of its usage and registration status. As shown in Figure 3, imidacloprid was been most extensively registered in China among the 10 NEOs. A review of GB 2763-2021 and the “National Standard for Food Safety Maximum Residue Limits for 112 Pesticides, including 2,4-D butyric acid sodium salt, in Foods” (GB 2763.1-2022) indicates that residue limits have been established for 84 pesticides in aquatic vegetables [19]. However, NEOs are not included in this list.

In this study, the detection rate of NEOs residues in *Oenanthe javanica* ranges from 3.5% to 44.76%. These results are consistent with those reported by other researchers noted in Table 4. In contrast, the 5th and 6th “National Total Diet Study” indicated detection rates of 53.3% and 70.5% for NEOs, suggesting that the detection rate obtained in this study is comparatively lower [13]. NEOs are novel, highly effective and low-toxicity insecticides. Meanwhile, NEOs are known for their long-lasting insecticidal activity and low toxicity to mammals. Consequently, they have been widely used for pest control instead of organophosphates, organochlorines and carbamates. However, recent studies indicate that NEOs are commonly found in various foods. Several NEOs (2–7) were found in 1280 samples from 24 provinces in China, with a detection rate of 37.58% [13]. In the present study, similar NEOs (2–6) were detected in 29 samples, with a lower detection rate of 20.28%. According to a study by Zhang [20], NEOs such as imidacloprid had a detection rate of 92% to 99% in 324 urine samples from 13 cities across China in 2019. Tu [21] also found NEOs and their metabolites in urine, blood and hair samples. These high detection rates and concentrations should be given attention by the government. Given regional differences in climate and environment, it is urgent to establish a pesticide registration system, promote the proper use of pesticides, enhance knowledge of safe application techniques in farmers, strengthen market regulation and develop residue limits for NEOs in *Oenanthe javanica*.

### 4.2. Dietary Risk Assessment Analysis

In this study, both acute and chronic dietary risk assessment methods were used to evaluate the risks of six NEOs (imidacloprid, thiamethoxam, acetamiprid, dinotefuran, clothianidin and flonicamid) for adults and children. The %*ADI* values for these pesticides ranged from 0.0034% to 0.58%, and the %*ARfD* values ranged from 0.01% to 2.86%, indicating that the acute and chronic dietary risks for adults and children were acceptable. However, acute risk was higher than chronic risk. Due to differences in body weight, children had higher %*ADI* and %*ARfD* values than adults, meaning the dietary risk for children was higher, consistent with Wei’s study [27].

The *HI* method and *RPF* approach were used to assess cumulative dietary risk for the same type of pesticides. The cumulative exposure values for children and adults were 0.87 and 0.26, respectively, and the combined exposure concentrations in the relative efficacy factor model were 0.00178 mg/kg/d and 0.00054 mg/kg/d. All these values are far lower than the health guidelines, indicating that the risks are acceptable. Wang [28] also found the maximum *HI* was 0.5187, which is below 1, indicating low risk, consistent with this study.

## 5. Conclusions

Neonicotinoid pesticide residues were commonly detected in fresh *Oenanthe javanica* sold in Hainan Province, with a detection rate of 49.65%, mainly consisting of imidacloprid. Dietary risk assessments indicated that the acute and chronic dietary intake risks of individual pesticides for all population groups were below 100%, while cumulative exposure risks were within the health guidance values. These findings suggest that the levels of NEOs in fresh *Oenanthe javanica* pose an acceptable risk to consumer health. This study provides a scientific basis for the safe consumption of *Oenanthe javanica* and the regulation of NEOs, offering critical technical support for the development and revision of relevant standards. In summary, it is crucial to establish pesticide registration systems and residue limits for NEOs in fresh *Oenanthe javanica* because of the wide use of NEOs. Meanwhile, it is also important to monitor the NEOs residues in food and carry out dietary risk assessments.

## Figures and Tables

**Table 1:** Relevant parameters of instrumental conditions.

NEOs	Retention Time (min)	Precursorion, *m*/*z*	Production, *m*/*z*	Linear Equation	*R* ^2^	Linear Range (mg/L)	Recovery Rates of Spiked (%)		RSD (%)	
							10 μg/kg	20 μg/kg	10 μg/kg	20 μg/kg
Imidacloprid	4.37	256.060	175.00/209.06	y = 15,437x + 31,974.5	0.9917	2–200	116	105	5.33	3.92
Thiamethoxam	3.91	292.030	181.10/211.07	y = 10,402x − 542.892	0.9978	2–200	102	97.5	5.78	7.26
Acetamiprid	4.67	223.070	99.00/126.00	y = 95,540.1x + 40,618.4	0.9931	2–100	106	101	0.39	2.27
Clothianidin	4.44	250.020	132.00/169.00	y = 15,403.6x + 1585.74	0.9955	2–200	97.4	94.6	1.37	1.85
Thiacloprid	4.96	253.030	99.00/126.00	y = 101,166x − 20,557.5	0.9954	2–50	119	120	0.61	1.34
Dinotefuran	3.35	203.100	129.10/157.10	y = 3217.7x − 517.721	0.9990	2–200	103	120	6.40	1.90
Nitenpyram	3.63	271.090	99.00/225.10	y = 4650.68x + 201.267	0.9982	2–200	109	104	0.71	6.01
Imidaclothiz	4.54	262.000	122.00/181.00	y = 13,330.1x + 12,302.8	0.9923	2–200	115	117	0.13	1.60
Flonicamid	3.87	230.000	148.05/203.07	y = 6388.57x + 57.1042	0.9973	2–200	119	105	8.39	3.57
Sulfoxaflor	4.75	278.032	153.87/173.89	y = 4536.22x–161.586	0.9966	2–100	108	105	3.87	0.75

**Table 2:** Multi-residue combinations of pesticide residues in *Oenanthe javanica* sold in Hainan Province.

Combination Count	Multi-Residue Combinations of Pesticides	Detection Number	Detection Rate (%)
Combination of Two	Imidacloprid/Acetamiprid	21	29.58
	Imidacloprid/Thiamethoxam	6	8.45
	Imidacloprid/Clothianidin	17	23.94
	Thiamethoxam/Acetamiprid	9	12.68
	Thiamethoxam/Clothianidin	10	14.08
	Acetamiprid/Clothianidin	14	19.72
Combination of Three	Imidacloprid/Thiamethoxam/Acetamiprid	6	8.45
	Imidacloprid/Thiamethoxam/Thiacloprid	6	8.45
	Imidacloprid/Thiamethoxam/Dinotefuran	3	4.23
	Imidacloprid/Acetamiprid/Clothianidin	14	19.72
	Imidacloprid/Thiamethoxam/Acetamiprid/Thiacloprid	6	8.45
Combination of Six	Imidacloprid/Thiamethoxam/Acetamiprid/Thiacloprid/Flonicamid/Dinotefuran	3	4.23

**Table 3:** *ADI*, *ARfD*, *cRfD* and *RPF* values of different NEOs and *RPF* values in different populations.

Insecticide Name	*ARfD* (mg/kg bw)	*ADI* (mg/kg bw)	Concentration (mg/kg)		*cRfD* (mg/kg/d)	*RPF*	Adult (mg/kg/d)		Children (mg/kg/d)	
			Highest Residue	Average			*BC_i_*	*TB_C_*	*BC_i_*	*TB_C_*
Imidacloprid	0.4	0.06	0.363	0.028	0.057	1.000	0.000149	0.000149	0.000491	0.000491
Thiamethoxam	1	0.08	0.084	0.0031	0.006	9.500	0.000016	0.000157	0.000054	0.000516
Acetamiprid	0.1	0.07	0.11	0.0073	0.071	0.803	0.000039	0.000031	0.000128	0.000103
Thiamethoxam	0.6	0.1	0.14	0.0053	0.01	5.700	0.000028	0.000161	0.000093	0.000530
Dinotefuran	1	0.2	0.012	0.0018	0.02	2.850	0.000010	0.000027	0.000032	0.000090
Flonicamid	0.025	0.07	0.024	0.002	0.04	1.425	0.000011	0.000015	0.000035	0.000050

**Table 4:** Research results on NEOs in food in recent years.

Year	Country	Sample	Number of Pesticides Monitored	Number of Pesticides Detected	Total Detection Rate (%)	Each Detection Rate (%)
2018	Chile [21]	Honey	4	4	18.8	6.3–18.8
2018	Japan [22]	Tea	7	7	100.0	3.0–100.0
2015	Ghana [23]	Cocoa	5	3	100.0	2.5–100.0
2019	China [24]	13 Everyday Foods	7	7	62.2	1.5–34.3
2018–2020	China [14]	Commercially available fruits and vegetables	3	3	80.0	12.0–55.1
2013	Spain [25]	Beeswax	7	3	36.7	1.3–26.7
2019	Slovenia [26]	Honey	4	2	60.8	11.7–58.9

## Data Availability

The original contributions presented in the study are included in the article, further inquiries can be directed to the corresponding authors.
